# Quantitative effect of subretinal fluid and intraretinal edema on visual acuity in uveitic cystoid macular edema

**DOI:** 10.1186/s12348-021-00266-y

**Published:** 2021-10-11

**Authors:** Eric W. Weldy, Jennifer L. Patnaik, Paula E. Pecen, Alan G. Palestine

**Affiliations:** Department of Ophthalmology, University of Colorado School of Medicine, 1635 Aurora Ct, Aurora, CO 80045 USA

**Keywords:** Uveitis, Cystoid macular edema, Subretinal fluid, Intraretinal edema, Optical coherence tomography

## Abstract

**Background:**

The effect of subretinal fluid (SRF) in uveitic cystoid macular edema (CME) is not fully understood. This study evaluates the quantitative effect of SRF and intraretinal thickness on visual acuity in eyes with uveitic CME. We separately measured SRF and intraretinal area on Optical Coherence Tomography (OCT) to determine the associations of each component with visual acuity and response to treatment.

**Main text:**

Medical records were reviewed of patients with CME presenting to the University of Colorado uveitis clinic from January 2012 to May 2019. All available OCTs were reviewed to classify eyes as either having only CME or CME with SRF. Intraretinal area was manually measured using Image J along the central 1-mm section of B-scan OCT spanning from the internal limiting membrane to the outer most portion of the outer retina including both cysts and retinal tissue. SRF cross-sectional area was measured spanning from the outermost portion of the outer retina to retinal pigment epithelium. Response to treatment was assessed one to four months after presentation. Eyes with CME secondary to structural or non-inflammatory causes were excluded.

Forty-seven (50.5%) eyes had CME alone and 46 (49.5%) eyes had SRF with CME. Measured SRF cross-sectional area was not associated (*p* = 0.21) with LogMAR at presentation. Conversely, intraretinal area was strongly correlated with visual acuity in eyes with SRF (*p* < 0.001) and without SRF (*p* < 0.001). Following treatment, there was a significant decrease in intraretinal area for both groups (*p* < 0.001), with a larger decrease in the SRF group compared to the non-SRF group (*p* = 0.001). Similarly, logMAR improved in both groups (*p* = 0.008 for SRF eyes and *p* = 0.005 for non-SRF eyes), but the change was more prominent in the SRF group (*p* = 0.06).

**Conclusions:**

There was no direct association observed between the amount of SRF and visual acuity. In contrast, increased intraretinal area was significantly associated with decreased visual acuity. This relationship between intraretinal thickening and visual acuity may explain differences observed in response to treatment between SRF and non-SRF eyes, with a larger decrease in the intraretinal cross-sectional area in SRF eyes associated with a greater improvement in logMAR visual acuity.

## Introduction

Uveitic macular edema is a common cause of vision loss [1, 2]. Nearly 40% of patients with non-infectious intermediate or posterior uveitis demonstrate structurally significant macular edema [3]. Despite its prevalence, the mechanism underlying extracellular fluid accumulation is unclear, although there is a presumed element of compromise to the blood-retinal barrier and retinal pigment epithelial pump function [4]. With optical coherence tomography (OCT), it is now feasible to accurately identify fluid collections as either cystoid macular edema (CME) due to intraretinal fluid, or as subretinal fluid (SRF) characterized by serous subfoveal detachment.

Previous studies have examined the clinical effect of retinal thickening in uveitic macular edema, demonstrating that central subfield thickness (CST) on OCT is moderately to strongly associated with visual acuity [5–9]. However, the effect of SRF on visual acuity is more challenging to directly measure and less well studied, despite it being a common finding in uveitic macular edema with a prevalence on OCT of 14–58% [5, 7–9]. Comprehensive reviews of uveitic macular edema have categorically shown that eyes with SRF tend to have overall worse vison at presentation and respond favorably to treatment [6, 10]. However, to our knowledge, there is no known study that quantitatively measures the SRF component directly to assess the extent of vision loss associated with SRF compared to intraretinal fluid.

Through manual measurement of intraretinal and subretinal fluid on OCT, the purpose of this study is to characterize the impact of each anatomic substrate on visual acuity and response to treatment.

## Methods

We conducted a retrospective review of patients with uveitic macular edema evaluated at the University of Colorado Department of Ophthalmology from January 2012 to May 2019. This retrospective study received Expedited IRB approval by the Colorado Multiple Institutional Review Boards, adhered to the Declarations of Helsinki, and informed consent was not required.

Patients with uveitic macular edema were identified through a query of the billing records for uveitis patients with cystoid macular edema presenting to the University of Colorado Eye Clinic’s two uveitis specialists (A.P., P.P.) during the study period. For each patient carrying this diagnosis, a review of all spectral-domain OCTs (Heidelberg Spectralis; Heidelberg Engineering, Heidelberg, Germany) during the study period were performed for both eyes. Patient eyes with macular edema on OCT secondary to an infectious, non-inflammatory, or structural etiologies other than uveitis were excluded (i.e. clinically significant epiretinal membrane, vein occlusion, choroidal neovascularization, vitreomacular traction, Irvine-Gass). Eyes with concurrent diabetic retinopathy or diabetic macular edema were also excluded. Patients with less than one-month follow-up and who did not follow up within at least 6 months after treatment were also excluded, as were eyes with dense media opacity that precluded accurate assessment of OCT features.

Cystoid macular edema was defined as any discrete hyporeflective pocket, regardless of size, seen on OCT within the retinal parenchyma. After reviewing all OCTs, patients with CME were classified as either having or not having SRF, which was defined as the presence of a subfoveal neurosensory retinal detachment at any timepoint in follow-up. If patients had multiple OCTs demonstrating SRF, the first visit in which SRF was identified was used as the baseline or “presentation” visit for inclusion in the SRF group. If no SRF was identified across all OCTs, the first visit at which CME was identified was used as baseline visit for the CME-only group. Eyes with macular thickening alone without CME were excluded.

Visual acuity was analyzed using logarithm of minimal angle of resolution (logMAR) transformation from visual acuity at baseline and at follow-up, which was determined as 1–4 months after the initial visit. In addition, the proportion of patients with acuities at standard thresholds (20/40 or better, 20/50 to 20/150, and 20/200 or worse) were reported. Age, sex, race/ethnicity, presence of diabetes, presence of associated systemic conditions, prior anti-inflammatory treatment (local steroid such as periocular and intravitreal agents, oral steroid, or immunomodulatory therapy), anti-inflammatory treatment at presentation, and anatomic location of uveitis (anterior, intermediate, posterior, or panuveitis) were also collected.

**Fig. 1:**
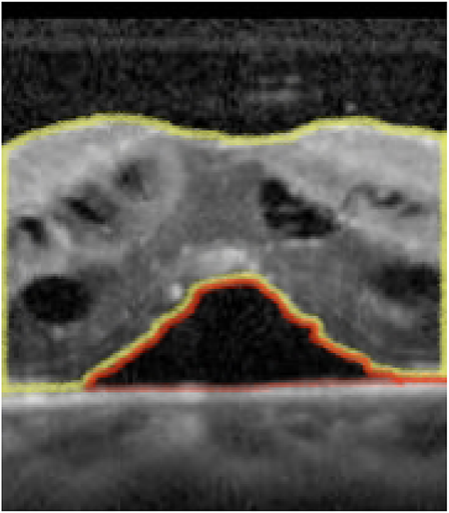
1-mm Cross-sectional OCT demonstrating manual measurements using Image J, including intraretinal area (outlined in yellow) and subretinal fluid area (outlined in red)

The intraretinal, subretinal and total retinal foveal cross-sectional area on OCT at presentation and follow-up was measured by a single investigator (EWW) using Image J software [11]. The foveal horizontal raster was uploaded for measurement and cropped to a total width of 1 mm centered on the fovea, which was accomplished by using the calipers intrinsic to the OCT software. Each component of interest in this OCT cross-section was measured via manual tracing accordingly: 1) intraretinal area measured from internal limiting membrane to the outer most portion of the outer retina including both cysts and retinal tissue (Fig. 1, area highlighted in yellow), 2) subretinal area measured from the outermost portion of the outer retina to retinal pigment epithelium (Fig. 1, area highlighted in red) and 3) total retinal area was a summation of the two measurements. This same process was repeated for the vertical foveal raster and values for all measurements were averaged. These OCT cross-sectional areas, which were proportional to volume, were used for the analysis.

### Statistical analysis

Eyes with SRF were compared to eyes without SRF using logistic regression modeling with generalized estimating equations to account for subjects who had two eyes included in the analysis. When there were cell sizes of zero, categories were combined for statistical testing. Spearman correlation coefficients were used to assess correlations between LogMAR and OCT measures. Linear models with generalized estimating equations with an unstructured correlation structure were used to compare LogMAR and cross-sectional areas at presentation and follow-up. Data were entered into Excel and SAS version 9.4 (Cary, NC) was used for all analyses.

## Results

Ninety-five patients with both non-infectious uveitis and CME on OCT were identified during the study period. Of these, 22 patients were excluded due to loss to follow up or missing OCTs (*n* = 16), dense media opacities (*n* = 3) or concurrent diabetic macular edema (n = 3). After exclusions there were 93 eyes of 73 patients for analysis, of which 47 (50.5%) eyes had CME alone and 46 (49.5%) eyes had SRF with CME. All eyes with SRF also had CME.

Demographic data and general characteristics of the study population are presented in Table 1. Eyes with SRF were compared to eyes without SRF, and there were no statistically significant differences in gender, anatomic location of uveitis and previous treatment history. Patient eyes with SRF were older, mean of 57 (SD 17.8) versus 45 years (SD 20.1), *p* = 0.02, and a higher proportion were Black compared to eyes without SRF (34.8% versus 4.3%, *p* = 0.007). SRF eyes were also more likely to have an underlying systemic condition (*p* = 0.05).

**Table 1:** Demographics of Eyes with Uveitic Cystoid Macular Edema, Comparing Eyes with and Without Subretinal Fluid at Presentation, *N* = 93

Characteristic	No Subretinal Fluidn = 47	Subretinal Fluidn = 46	***p***-value
Gender			0.64
Male	22 (46.8%)	19 (41.3%)	
Female	25 (53.2%)	27 (58.7%)	
Age, Mean (SD), Years	45.2 (20.1)	57.0 (17.8)	0.02
Race			0.007
White	41 (87.2%)	28 (60.9%)	
Black	2 (4.3%)	16 (34.8%)	
Hispanic*	2 (4.3%)	2 (4.4%)	
Asian*	2 (4.3%)	0 (0%)	
Location, No. (%) of patients			0.84
Anterior	4 (8.5%)	5 (10.9%)	
Anterior and Intermediate	3 (6.4%)	5 (10.9%)	
Intermediate	25 (53.2%)	20 (43.5%)	
Panuveitis**	13 (27.7%)	16 (34.8%)	
Posterior**	2 (4.3%)	0 (0%)	
Origin, No. (%) of patients			0.05
Associated Systemic Disease	3 (6.4%)	2 (4.4%)	
HLA-B27	2 (4.3%)	3 (6.5%)	
Multiple Sclerosis	0 (0%)	2 (4.4%)	
Sarcoidosis	0 (0%)	6 (13.0%)	
Undifferentiated***	42 (89.4%)	33 (71.7%)	
Treatment prior to presentation****			
Local steroids	14 (29.8%)	12 (26.1%)	0.71
Systemic steroids	5 (10.6%)	3 (6.5%)	0.57
Immunomodulatory Therapy	12 (25.5%)	10 (21.7%)	0.69
Treatment at presentation****			
Local steroids	34 (72.3%)	32 (69.6%)	0.79
Systemic steroids	1 (2.1%)	2 (4.4%)	0.55
Immunomodulatory Therapy	4 (8.5%)	0 (0%)	0.12*****

*Asian race and Hispanic ethnicity combined for statistical testing

**Panuveitis and Posterior combined for statistical testing

***Undifferentiated versus all other origins combined for statistical testing

****Eyes could have had more than one type of treatment

*****Fisher’s exact testing because of zero cell size

Visual acuity at presentation was slightly worse in those with SRF compared to those with CME alone (median logMAR 0.477 and 0.398, respectively), but this difference was not statistically significant (*p* = 0.73, Table 2). However, when VA was categorized into groups of 20/40 or better, 20/50–20/150, or 20/200 or worse, eyes with and without SRF had a different distribution across the VA categories (*p* < 0.001). Eyes with SRF had a higher proportion of moderate vision loss at presentation, 87% (40/46) had visual acuity 20/50–20/150, vs 47% (22/47) of eyes with CME alone. Eyes without SRF had a slightly higher rate of severe vision loss (12.8% versus 2.2%).

**Table 2:** Visual acuity in Eyes with Uveitic Cystoid Macular Edema, Comparing eyes with and without Subretinal Fluid. *N* = 93

Variable	No Subretinal Fluid	Subretinal Fluid	p-value
Visual acuity at presentation LogMAR, median (range)	0.398 (0–1.602)	0.477 (0–1.301)	0.73
Visual acuity by category at presentation, No. (%)			< 0.001
20/40 or better	19 (40.4%)	5 (10.9%)	
20/50–20/150	22 (46.8%)	40 (87.0%)	
20/200 or worse	6 (12.8%)	1 (2.2%)	
Visual acuity at follow up			0.28
LogMAR, median (range)	0.301 (0–1.602)	0.238 (0–1.000)	
Visual acuity by category at follow-up, No. (%)			0.54
20/40 or better	31 (66.0%)	30 (65.2%)	
20/50–20/150	13 (27.7%)	15 (32.6%)	
20/200 or worse	3 (6.4%)	1 (2.2%)	
Change in LogMAR			0.06
Median (range)	−0.125 (−0.301–0.574)	−0.213 (− 0.602–1.0)	

Eyes were categorized by presence versus absence of subretinal fluid based on initial presentation.

OCT measurements are presented in Table 3. At presentation, initial intraretinal cross-sectional area in SRF eyes was slightly higher compared to eyes with CME alone, although this difference was not statistically significant (*p* = 0.18). Total retinal cross-sectional area at presentation was greater in eyes with SRF than the nonsubfoveal SRF group (*p* = 0.003)

**Table 3:** Cross-sectional area of OCT structures in Eyes with Uveitic Cystoid Macular Edema, Comparing Eyes with and without Subretinal Fluid. *N* = 93

Variable	Eyes without Subretinal Fluid	Eyes with Subretinal Fluid	***p***-value
At presentation			
Intraretinal area, median (range), mm^2^	0.42 (0.29–0.75)	0.44 (0.23–0.69)	0.18
Subretinal Fluid Area, median (range), mm^2^	N/A	0.026 (0.0029–0.29)	N/A
Total Retinal Area, median (range), mm^2^	0.42 (0.29–0.75)	0.51 (0.30–0.95)	0.003
At follow -up			
Intraretinal Area, median (range), mm^2^	0.33 (0.24–1.11)	0.32 (0.24–0.74)	0.90
Subretinal Fluid Area, median (range), mm^2^	N/A	0.011 (0.003–0.30)*	N/A
Total Retinal Area, median (range), mm^2^	0.33 (0.24–1.11)	0.33 (0.24–0.77)	0.49

Abbreviations: N/A Not applicable, OCT Optical Coherence Tomography

*Includes only eyes with persistent subretinal fluid, which was 12 of 46 eyes

.

Eyes were categorized by presence versus absence of subretinal fluid based on initial presentation.

In SRF eyes, the median cross-sectional area of subfoveal subretinal fluid was 0.026 mm^2^. There was no correlation between intraretinal thickening and the amount of subretinal fluid in SRF eyes (Spearman correlation coefficient 0.19, *p* = 0.21). However, when comparing visual acuity among quartiles of subretinal fluid, the uppermost quartile for SRF had worse vision compared to other SRF eyes (*p* = 0.02), despite having a similar intraretinal cross-sectional area (*p* = 0.873).

**Fig. 2:**
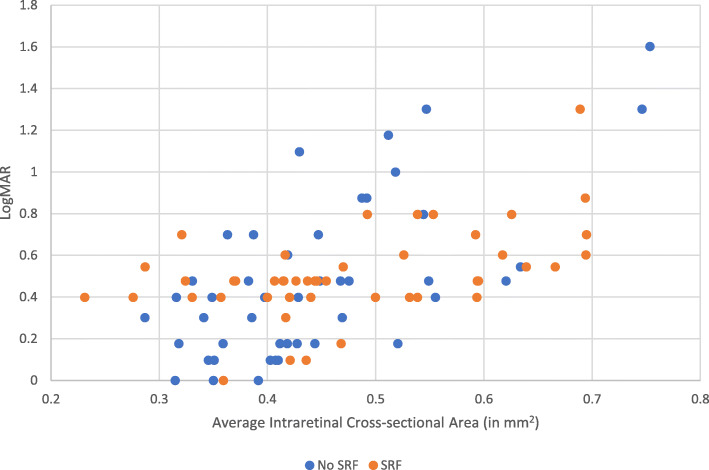
Plot of average intraretinal cross-sectional area by LogMAR for eyes with and without SRF at presentation. The intraretinal cross-sectional area was strongly correlated to visual acuity in eyes with SRF (Spearman correlation coefficient 0.49, *p* < 0.001) and eyes without SRF (Spearman correlation coefficient 0.59, *p* < 0.001)

The intraretinal cross-sectional area was correlated with visual acuity in eyes with SRF (Spearman correlation coefficient 0.49, *p* < 0.001) and even more so for eyes without SRF (Spearman correlation coefficient 0.59, p < 0.001) (Fig. 2). Using univariate analysis modeling of all eyes with LogMAR at presentation as the outcome, both the initial intraretinal (p < 0.001) and total retinal cross-sectional area (p = < 0.001) measured at presentation were significantly associated. In contrast, SRF cross-sectional area was not associated (*p* = 0.21) with LogMAR at presentation.

Treatment of the cases and controls prior to presentation did not differ (Table 1). There were no differences in history of local steroid therapy (i.e. periocular and intravitreal agents), systemic steroid therapy, or immunosuppressive therapy (*p* = 0.71, *p* = 0.57, *p* = 0.69, respectively). Similarly, treatment at presentation of cases and controls did not differ, with comparable rates of local steroid therapy, systemic steroid therapy, and immunosuppressive therapy (*p* = 0.79, *p* = 0.55, *p* = 0.12, respectively).

Follow-up visual acuity one to four months after treatment with anti-inflammatory agents is presented in Table 2. LogMAR from presentation to follow-up improved in both groups (*p* = 0.008 for SRF eyes and *p* = 0.005 for non-SRF eyes). At follow up, there was no difference in visual acuity between the SRF and non-SRF groups (*p* = 0.28). In contrast to presentation, analysis of VA as categories showed similar distribution between the two groups (*p* = 0.54) and similar rates of moderate vision loss, 15/46 (32.6%) with SRF versus 13/47 (27.7%) with CME alone.

At follow-up, the median total retinal cross-sectional area decreased from 0.51 mm^2^ to 0.33 mm^2^ in the SRF group (*p* < 0.001) and 0.42 mm^2^ to 0.33 mm^2^ in the group without SRF (*p* < 0.001, Table 3). The decrease was more prominent in the SRF group (*p* = 0.001), such that at follow up, there was no difference (*p* = 0.49). The median intraretinal cross-sectional area at presentation was 0.44 mm^2^ in the SRF group and 0.42 mm^2^ in the non-SRF group (*p* = 0.18). There was a significant decrease for both groups (p < 0.001 in SRF eyes, p < 0.001 in non-SRF eyes), with a larger decrease in the SRF group (p = 0.001). Thirty-four eyes (73.9%) with SRF at presentation showed complete resolution of SRF at follow-up. For the remaining twelve eyes with persistent SRF, the median cross-sectional area was 0.011 mm^2^.

## Discussion

Previous studies have reported that uveitic eyes with SRF categorically have worse initial visual acuity associated with greater central subfoveal thickness (CST) when compared to eyes without SRF [5, 6, 10]. Despite these differences, SRF eyes have been shown to respond favorably to treatment to obtain similar visual acuity and retinal thickness at follow-up, a finding observed in our study as well.

In measuring SRF cross-sectional area directly on OCT, our study is the first to quantitatively report that there is no direct association between the amount of SRF and visual acuity. As an exception to this observation, when comparing visual acuity among quartiles of subretinal fluid, we found that the uppermost quartile for SRF had worse vision compared to other SRF eyes, despite having a similar degree of intraretinal thickening. This observation suggests there may be a threshold volume of subretinal fluid, above which visual acuity is negatively affected.

In contrast, linear regression and correlation analysis demonstrated that increased intraretinal cross-sectional area was significantly associated with decreased visual acuity. This relationship between intraretinal thickening and visual acuity may explain differences observed in response to treatment between SRF and non-SRF eyes. For example, a larger decrease in the intraretinal cross-sectional area was observed among SRF eyes, and SRF eyes also had a greater improvement in logMAR VA.

These findings are distinct from previous studies that used total retinal thickness (TRT) to correlate with visual acuity. Tran et al [7] compared the correlation between TRT and vision in eyes with and without SRF, demonstrating that SRF eyes had a weaker correlation to VA compared to eyes with CME alone. Tran et al. suggested that subretinal fluid volume be subtracted from central thickness measurements when correlating with vision. Sivaprasad et al. found no association between CST or VA in SRF eyes [9]. More recently, Ossewaarde et al [6] found an association between TRT and VA independent of SRF presence.

However, in using TRT, the above studies do not directly examine the subretinal fluid component itself. Our data showed there was no correlation between the degree of intraretinal edema and the amount of subretinal fluid in SRF eyes, implying it is possible to have predominantly intraretinal thickening with minimal SRF and vice versa. Since total retinal thickness does not necessarily correlate with either anatomic component on OCT, CST is inadequate for assessing extent of SRF. Therefore, defining the impact of subretinal fluid requires direct measurement of each component of CST.

Ossewaarde et al [6] did measure the height of SRF in uveitic eyes to identify an association between SRF height and vision. However, this approach does not reflect that SRF may assume a variety of irregular contours beyond a single subfoveal point. A correlate to the 1 mm diameter subfoveal CST, as was done in our study, more reliably captures the relationships between intraretinal edema, SRF and visual acuity.

It is unclear as to why the intraretinal fluid component causes vision loss in uveitic eyes. One hypothesis is that intraretinal fluid causes disorganization of photoreceptors, compromising the optical properties and directional sensitivity of retinal cells [12]. Similarly, in both age-related macular degeneration (AMD) and diabetic macular edema (DME) there is a strong negative correlation between intraretinal thickening and visual acuity [13, 14]. However, the mechanism by which intraretinal fluid accumulates may differ compared to uveitic CME, evidenced by the fact that exudates are virtually never seen in uveitis, whereas they are commonly seen in DME and often in AMD. As for SRF, vision loss was found in patients with larger amounts of subretinal fluid in our study. This suggests there may be a threshold beyond which cell renewal or maintenance of metabolic function by the retinal pigment epithelium is impaired. This is in contrast to SRF in AMD which has been associated with favorable initial visual acuity [13] and SRF in DME which has been associated with categorically worse visual acuity and response to treatment [15, 16].

Several limitations must be noted. The sample size is fairly small, and as such there may be a relationship between visual acuity and subretinal fluid that we are unable to detect. However, our sample size is similar to previous literature. Furthermore, our results clearly identify several important statistically significant differences and provide a foundation for hypothesis generation and analysis of larger cohorts of uveitic CME. Our study results may not be generalizable to all patients with uveitis. Our institution is a tertiary referral center for uveitis and the possibility of referral bias must be acknowledged. For those seen at our institution, we were able to review all OCTs for patients with the diagnosis of uveitic macular edema during the study period to limit selection bias. Furthermore, the results of our statistical analysis are limited to eyes without other contributing causes of macular edema. The rationale for this is to allow clear interpretation of the relationships between uveitic intraretinal and subretinal fluid and visual acuity, while limiting confounding. As such, associations found within the context of this study may not apply to uveitic eyes with concurrent vitreoretinal pathology. Additionally, this study also did not evaluate for the presence of other factors associated with uveitis that may contribute to vision loss, such as anterior or posterior segment inflammation or media opacities.

Overall, we found that 50% of uveitic eyes with OCT-proven macular edema had subfoveal SRF at any point during the study period. This is comparable with previous studies, reporting a prevalence ranging from 14% to 58% [5–10]. Since our study defined presence of SRF as the first occurrence during the study period of 7 years, this may reflect a higher incidence in the context of longer term follow up with frequent OCT examination for some patients.

In conclusion, our study provides insight into vision loss in uveitis patients with SRF, a relatively common OCT finding in uveitic macular edema. We showed that vision loss is strongly associated with the intraretinal thickening, as opposed to subretinal fluid. However, large amounts of subretinal fluid are associated with worse vision as well. Newer OCT models with integrated volumetric analysis software should be used to confirm these relationships as this technology becomes more readily available.

## Data Availability

The datasets used and/or analyzed during the current study are available from the corresponding author on reasonable request.

## Abbreviations

OCT: Optical coherence tomography
CME: cystoid macular edema
SRF: subretinal fluid
CST: central subfield thickness
TRT: total retinal thickness
VA: Visual acuity
